# 

**Published:** 1919-09-27

**Authors:** 


Mr. David Halley, of Crieff, has bequeathed £1,000 each to the Dundee Royal Infirmary and the Edinburgh Royal Infirmary, and about £3,000 in smaller amounts to other charitable institutions.

### The College of Nursing (Limited by Guarantee).

SHEFFIELD CENTRE.

A meeting of members of the College was held at the Royal Infirmary on Monday, September 22. In spite of the very bad weather the attendance was good. The names of thirteen new members were handed in, and much enthusiasm and interest in the progress of the Sheffield Branch was displayed. The Chair of Nursing was discussed, and all present voted in favour of the scheme. The arrangements for the Winter Session include a Post-Graduate Course of Lectures, commencing early in October, and a Sale of Work in the New Year for the funds of the local Centre. The Secretary will be glad if members will notify her of any change of address, as cards for the lectures are being sent out.

BRIGHTON AND HOVE CENTRE.

Mrs. Scharlieb's Lecture.

An overflowing audience assembled to hear the lecture by Dr. Mary Scharlieb on Venereal Diseases which was given at this Centre last week. The chair was taken by Dr. Helen Boyle, and amongst the audience were several members of the medical profession. Refreshments were served after the lecture.

Residential Club.

The arrangements for this development of the Brighton Centre are proceeding apace, and the Club will be open to receive guests in the third week of October. Sleeping accommodation is provided for members of the College of Nursing only. They will be able to have lunches, teas, or suppers at very moderate charges. Members will have the right to entertain friends. There are already many requests for rooms, and members of the College who wish to spend a holiday in Brighton are advised to make early application.

The Bazaar.

The Bazaar is to be held the first week in December, and has secured the help of the whole country. The Hon. Secretary will be delighted to receive either donations or gifts towards it.

### Lectures on Tuberculosis.

AUTUMN SESSION.

The following lectures will be given at the Hospital for Consumption and Diseases of the Chest, Brompton, S.W. 3, at 8 P.M. :—

**Oct. 21.**—1. "The History of Tuberculosis," L. S. T. Burrell, M.A., M.D. M.R.C.P **Oct. 24.**—2 "The Cause of Tuberculosis," A. C. Inman, M.A., M.B. **Oct. 28.**—3. "Tuberculosis as an Infective Disease," A. C. Inman, M.A, M.B. **Oct. 31.**—4. "Its Distribution and Incidence," L. S. T. Burrell, M.A., M.D., M.R.C.P. **Nov. 4.**—5. "The Human Body as Possible Host," T. Gwynne Maitland, M.A., B.Sc., M.D, D.Phil. **Nov. 7.**—6. "The Biological Aspects of Tuberculosis," A. C. Inman, M.A., M.B. **Nov. 11.**—7. "The Pathological Anatomy of Tuberculosis," A. C. Inman, M.A, M.B. **Nov. 14.**—8. "The Nose and Throat in Relation to Tuberculosis," J. Dundas Grant, M.D., F.R.C.S. **Nov. 18.**—9. "The X-Ray Evidence," Stanley Melville, M.D. **Nov. 21.**—10. "Pulmonary Tuberculosis, Clinical," A. J. Jex-Blake, M.A., M.B., F.R.C.P. **Nov. 25.**—11. "Surgical Tuberculosis," L. S. T. Burrell, M.A., M.D., M.R.C.P. **Nov. 28.**—12. "The Circulatory System," A. Hope Gosse, M.A., M.D, M.R.C.P. **Dec. 2.**—13. "The Nursing of Tuberculosis, General," T. Gwynne Maitland, M.A., B.Sc., M.D., D.Phil. **Dec. 5.**—14. "The Home Nursing of Tuberculosis," Miss F. T. Redl **Dec. 9.**—15. "Sanatorium Treatment, and After," W. O. Meek, M.B., M.R.C.S. **Dec. 12**—16. "Economic Factors," Miss Gladys Bompas. **Dec. 16.**—17. "Present and Future," T. Gwynne Maitland, M.A., B.Sc., M.D., D.Phil.

The Fee for the Course is **£1 1s.** Lectures, with practical work and optional examination and certificate to successful candidates, **£2 2s.**

